# Basic Protein Modules Combining Abscisic Acid and Light Signaling in *Arabidopsis*

**DOI:** 10.3389/fpls.2021.808960

**Published:** 2022-01-03

**Authors:** Victor P. Bulgakov, Olga G. Koren

**Affiliations:** Department of Biotechnology, Federal Scientific Center of the East Asia Terrestrial Biodiversity, Far Eastern Branch of the Russian Academy of Sciences, Vladivostok, Russia

**Keywords:** ABA signaling, *Arabidopsis*, light signaling, interactome, protein modules, stress response, growth and development

## Abstract

It is generally accepted that plants use the complex signaling system regulated by light and abscisic acid (ABA) signaling components to optimize growth and development in different situations. The role of ABA–light interactions is evident in the coupling of stress defense reactions with seed germination and root development, maintaining of stem cell identity and stem cell specification, stem elongation and leaf development, flowering and fruit formation, senescence, and shade avoidance. All these processes are regulated jointly by the ABA–light signaling system. Although a lot of work has been devoted to ABA–light signal interactions, there is still no systematic description of central signaling components and protein modules, which jointly regulate plant development. New data have emerged to promote understanding of how ABA and light signals are integrated at the molecular level, representing an extensively growing area of research. This work is intended to fill existing gaps by using literature data combined with bioinformatics analysis.

## Introduction

Several years ago, Lau and Deng (2010) summarized data on the interaction of light signaling pathways and phytohormones and noted that phytochrome-interacting factors PIF3, PIF4, PIF1/PIL5, and HY5 (long hypocotyls 5) are main light signaling components that link light signals to the signaling of phytohormones. At this time, the role of abscisic acid (ABA) signaling in light response was still poorly understood. Presently, many aspects of the interaction of light and ABA signaling were considered, in particular ABA involvement in the circadian clock (Seung et al., 2012), flowering (Park et al., 2016), guard cell sensory system (Assmann and Jegla, 2016), chlorophyll metabolism (Zhu et al., 2017), shade avoidance (Courbier and Pierik, 2019), flavonoid biosynthesis (Brunetti et al., 2019), and seedling development (Yadukrishnan and Datta, 2021). These articles mainly describe interactions of the light signaling system with ABI5 (protein abscisic acid-insensitive 5), an important signaling hub in the ABA pathway.

Although many articles consider particular aspects of cooperation between ABA and light signaling, there are no works that summarize all protein modules involved in the cooperation. For the description of the ABA–light signaling system in *Arabidopsis thaliana* (*Arabidopsis*), we used a systematic approach based on the analysis of literature data, databases, and analysis of protein interaction networks. By analyzing these data, we determined which *Arabidopsis* protein modules connect ABA and light signaling components. We created an integral picture of known interactions but left aside the signaling of phototropins, cryptochromes, and ultraviolet-B receptor UVR8 because their modules are still poorly resolved. Brassinosteroid (BR) and ethylene (ET) signaling were not considered in this work, although they participate in the regulation of ABA–light interplay. The reason for this is that the combined system BR–ET–ABA–light is very cumbersome to visualize and difficult to understand. Thus, this article describes only ABA–light interactions. We concentrated mainly on the balance between stress reactions and growth mediated jointly by the ABA–light signaling system. A recent review article by Yadukrishnan and Datta (2021) focused on ABA and light signaling in early seedling development. The authors noted that light causes a counteractive effect on ABA signaling, but their interaction cannot be considered as opposing each other. We are also discussing these aspects. Protein names are presented as recommended by the UniProtKB database.

## ABA Signaling

In non-stress conditions, ABA plays a dual role, acting as an inhibitor or activator of plant growth and development. ABA promotes hypocotyl elongation in the dark, promotes leaf and root growth as well as inhibiting stomatal transpiration and leaf initiation (Yoshida et al., 2019). ABA under non-stress conditions is important for correct stomatal development and floral transition (Yoshida et al., 2019). Under stress conditions, ABA controls stomatal aperture, thereby reducing CO_2_ entry into leaves and limiting photosynthesis and increasing tolerance to stress conditions (Dong et al., 2015; Kuromori et al., 2018). Endogenous basal ABA does not directly suppress photosynthesis and inhibits a stress-escape response (avoidance of stress through reproduction, a response that is unfavorable for an individual plant, but necessary to preserve the species) under normal conditions, allowing plants to accumulate biomass and increase yield (Negin et al., 2019). However, long-term ABA treatment causes COP1 activation in a light intensity-dependent manner to suppress chloroplast development and balance growth and stress responses (Lee et al., 2021). PhyB stimulates the ABA biosynthesis in the shoots, and mobile shoot-to-root ABA signaling links phyB-mediated light perception with root reactive oxygen species (ROS) homeostasis for optimal plant propagation (Ha et al., 2018).

**Figure 1:**
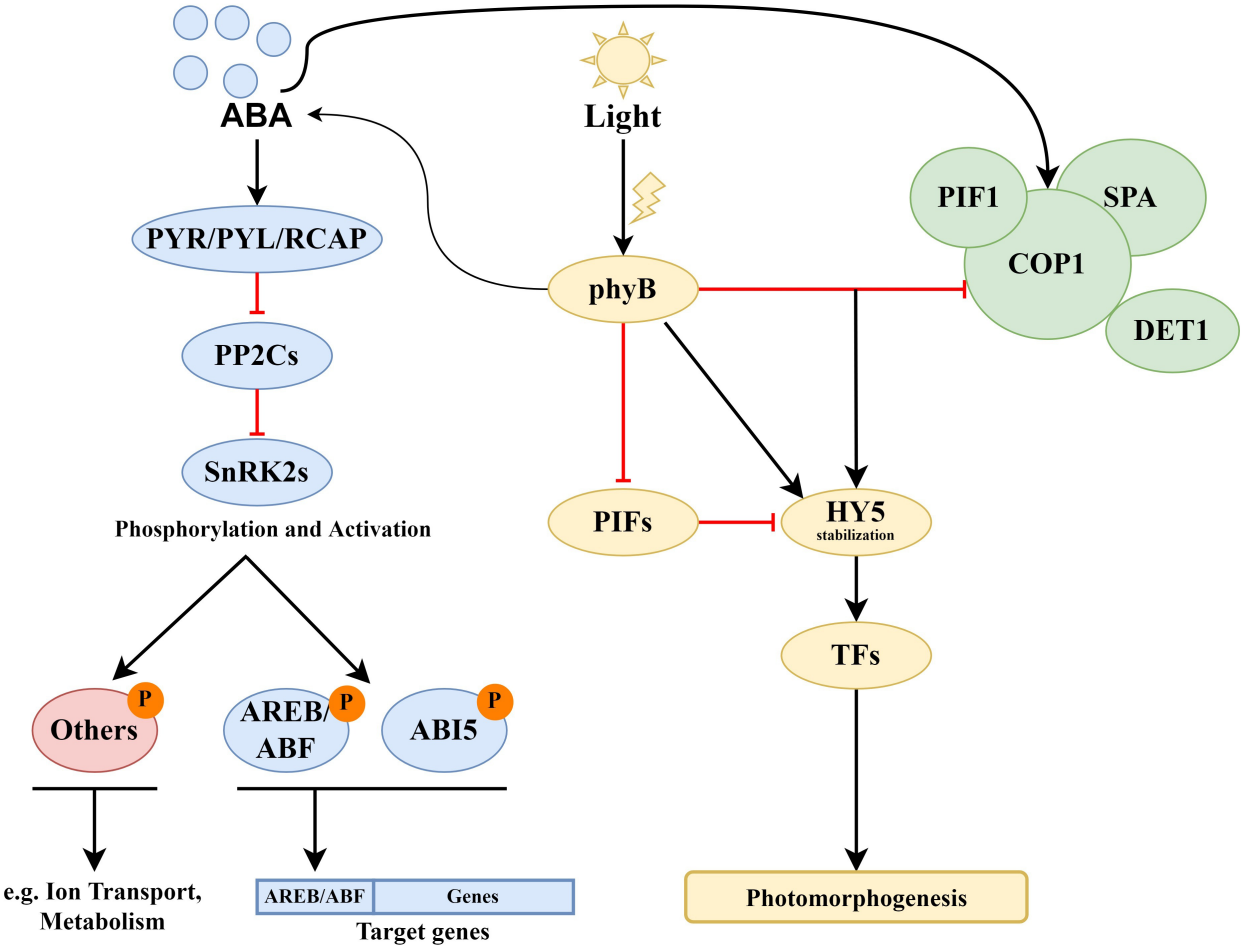
Core components of the ABA and light signaling systems. Long-term ABA treatment causes COP1 activation and phyB stimulates the ABA biosynthesis. ABA activates ABA receptors of the PYR/PYL/RCAR family that inhibits A-type protein phosphatases 2C (PP2Cs). PP2C inactivation activates class 3 sucrose nonfermenting-1-related protein kinases (SnRK2s) that phosphorylate ABA-responsive element binding factors AREB/ABF. Activated ABFs initiate the expression of responsive genes by binding to the *cis*-acting ABA response element (ABRE). In response to light, phytochromes inactivate COP1-SPA complexes and induce degradation of negative regulators of photomorphogenesis, PIFs. PhyB inhibits the regulatory activity of PIF1 and PIF3. These events promote photomorphogenesis by establishing the dynamic protein module PIF1-COP1/SPA1 and triggering HY5-mediated light signaling. Degradation of PIFs and stabilization of HY5 result in promotion of photomorphogenesis. HY5 physically interacts with transcription factors (TFs) and other proteins, and directly affects the expression of numerous genes. By promoting COP1 protein destabilization and turnover, DET1 positively regulates COP1 activity toward the degradation of HY5. Keeping HY5 levels tightly regulated is essential for its functioning during dark-to-light transition.

Stress induces ABA accumulation and binding to ABA receptors of the PYR/PYL/RCAR family to inhibit A-type protein phosphatases 2C (PP2Cs). PP2C inactivation activates class 3 sucrose nonfermenting-1-related protein kinases (SnRK2s) that phosphorylate ABA-responsive element binding factors (ABFs). Activated ABFs initiate the expression of responsive genes by binding to the *cis*-acting ABA response element (ABRE, Fujii et al., 2009; Lumba et al., 2014). The basic scheme of ABA signaling is shown in Figure 1.

## Basic Photomorphogenetic Mechanisms

The *A. thaliana* genome encodes five phytochrome proteins: phyA mediates responses to far-red light (FR), and phyB-phyE mediates shade avoidance and classical red light (R)/FR-reversible responses (Clack et al., 2009). In the dark, plants repress photomorphogenesis by the complexes consisting of E3 ubiquitin-protein ligase COP1 (constitutive morphogenic1) and SPA (suppressor of phytochrome A) proteins (Xu et al., 2014). An important role in the degradation of positively acting transcription factors is also played by basic helix–loop–helix (bHLH) transcription factors PIFs. In response to light, phytochromes inactivate COP1-SPA complexes and induce degradation of negative regulators of photomorphogenesis, PIFs (Figure 1). PhyB inhibits the regulatory activity of PIF1 (synonym: PIL5) and PIF3 by releasing them from DNA targets. These events promote photomorphogenesis by establishing the dynamic protein module PIF1-COP1/SPA1 and triggering HY5-mediated light signaling (Xu et al., 2014; Pham et al., 2018a; Paik et al., 2019; Yang and Liu, 2020). The COP1-SPA complexes are also important for sensing vegetative shade, which is determined as a reduction in the R:FR ratio and is essential for hypocotyl growth and leaf petiole elongation.

PIF1, PIF3, PIF4, PIF5, and PIF7 contain an active phyB-binding (APB) domain, which is necessary for interaction with light-activated phyB. PIF1 and PIF3 also bind to phyA through an additional phyA-binding (APA) domain (Legris et al., 2017). According to Xu et al. (2014), the bHLH domain of PIF1 interacts with the bZIP domain of HY5, and the N-terminal domain of PIF1 interacts with the WD40 repeat domain of COP1. The resulting complex promotes degradation of HY5 *via* the 26S proteasome pathway. PIF4 is an integrator of numerous signaling pathways and optimizes growth in environmental conditions (Choi and Oh, 2016; Legris et al., 2017). PIF4 is better known as a regulator of thermosensitive growth, although its role in cold resistance is also known (Choi and Oh, 2016). The role of PIFs as critical signal integrators is discussed in recent review articles (Legris et al., 2017; Paik et al., 2017; Pham et al., 2018a,b; Favero, 2020; Jing and Lin, 2020).

## ABA–Light Integrative Modules

**Figure 2:**
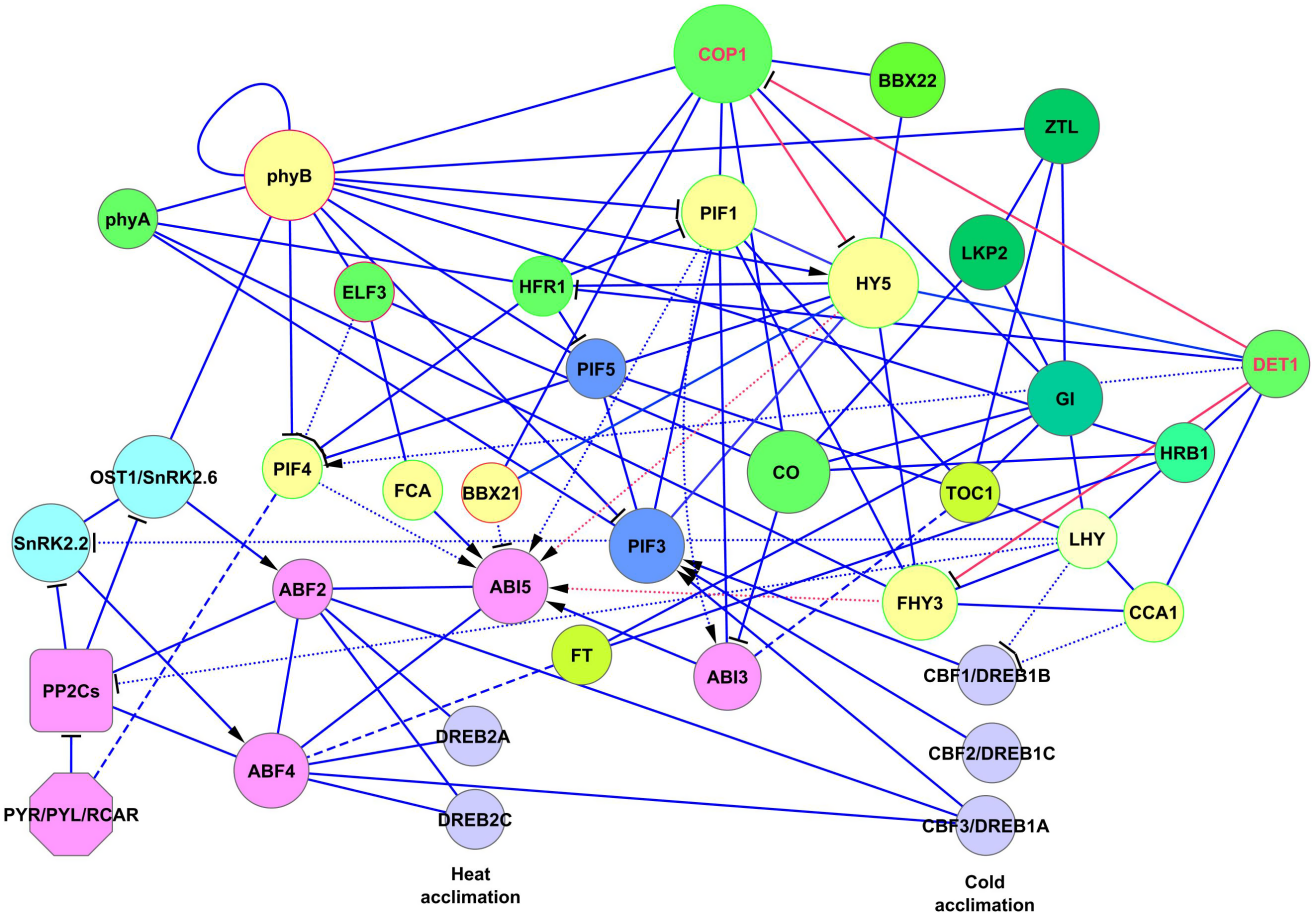
General presentation of the ABA–light signaling network. Yellow circles represent proteins of the light signaling system, physically interacting with ABA proteins (solid lines) or affecting gene expression (dotted lines). Dashed lines represent protein–protein interactions with unknown functions. Other proteins in the light signaling network are shown in green, while ABA proteins are in pink and blue. CBF/DREB proteins are presented in lilac color. Interactions were visualized using the Cytoscape program. The data loaded into the program were obtained from the PAIR database. The protein–protein interactions presented in PAIR were verified using the data from BioGRID, UniProtKB, TAIR, and IntAct databases. Abbreviations: PYR/PYL/RCAR, ABA receptors of the PYR/PYL/RCAR family, PP2Cs, A-type PP2Cs; ABFs, abscisic acid responsive elements-binding factors; ABI3/5, ABA insensitive 3/5; OST1/SnRK2.6, SNF1-related protein kinase 2.6; SnRK2.2, SNF1-related protein kinase 2.2; phyA/B, phytochromes phyA and phyB, PIFs, phytochrome-interacting transcription factors; HY5, transcription factor HY5 (synonym: long hypocotyls 5); CO, CONSTANS; FCA, flowering time control protein; FHY3, far-red elongated hypocotyls 3; BBX21/22, B-box zinc finger transcription factors BBX21 and BBX22; DET1, light-mediated development protein; HRB1, dehydration-induced 19 homologs 7 HRB1/DI19-7; ELF3, early flowering 3; TOC1, two-component response regulator-like APRR1 (synonym: timing of CAB expression 1); FT, flowering locus T; HFR1, transcription factor HFR1 (synonym: long hypocotyl in far-red 1); COP1, E3 ubiquitin-protein ligase COP1 (synonym: constitutive photomorphogenesis 1); LHY, protein late elongated hypocotyl; CCA1, circadian clock associated 1; GI, GIGANTEA; LKP2, adagio protein 2; ZTL, adagio protein 1; DREBs, dehydration-responsive element-binding proteins.

Figure 2 represents the joint ABA–light signaling network generated on the base of the *Arabidopsis* PAIR database (Lin et al., 2011).^1^^,^^2^^,^^3^^,^^4^^,^^5^ The ABA receptors PYR/PYL/RCAR and phyA/B were chosen as input proteins for the network. Yellow circles represent proteins of the light signaling system, which has a function in the Gene Ontology (GO) Process “Response to Abscisic Acid.” Each of these proteins forms its own signaling module, described in separate subsections of the article. The most known and probably most important module is based on HY5. Far-red elongated hypocotyls 3 (FHY3) and far-red impaired response 1 (FAR1) are positive regulators of ABA signaling that participate in plant development, The MYB-related transcription factors LHY/CCA1 serve as a link between the circadian clock and cold response. The main role of early flowering 3 (ELF3), as a component of the Evening Complex, is to connect light signaling with thermotolerance. Flowering time control protein FCA (FCA) is involved in the ABA pathway and ROS modulation (Lee et al., 2015), and transcription factor HFR1 is involved in the response to light intensity, shade avoidance, and dark-induced senescence. The ABI3-CONSTANS (CO) interaction has a role in the maintenance of embryo dormancy (Kurup et al., 2000). These proteins and interactions between them are critical for interaction with the ABA signaling system. In addition, the data indicate several interactions between ABA and light-signaling proteins with as yet unknown physiological roles. Among them are the flowering locus T (FT)-ABF4 interaction (Braun et al., 2011), ABI3-TOC1 (Kurup et al., 2000), and interactions of ABA receptors with PIFs (Qi et al., 2020). ABA–light integrative modules and descriptions of their functional significance are presented in Table 1.

**Table 1:** ABA–light integrative modules and their functional significance.

Light signaling protein	ABA-responsive gene/protein	Signaling module	Function	References
HY5	*ABI5*	HY5 → *ABI5*	ABA responses in seed germination, seedling growth, and root development	Chen et al., 2008
			H_2_O_2_ production *via* the activation of NADPH oxidase. Tolerance to excess light and cold	Zhou et al., 2014; Wang et al., 2018
BBX21	*ABI5*	BBX21—‖ *ABI5*	Gas exchange in drought conditions	Xu et al., 2014; Kang et al., 2018
PIF1	*ABI3* and *ABI5*	phyB —‖ PIF1 → *ABI3* and *ABI5*	Increased ABA responsiveness, inhibition of seed germination	Oh et al., 2009
PIF1 PIF4	*ABI5*	ELF3 —‖ *PIF4* → *ABI5*	Leaf senescence at high ambient temperature	Sakuraba et al., 2014; Kim et al., 2020
PIF1 PIF3 PIF4 PIF5	PYL8/PYL9	PYL8/PYL9 → PIF4	PYL8 and PYL9 promote PIF4 protein accumulation in the dark and enhance PIF4 binding to the *ABI5* promoter, but negatively regulate PIF4-mediated *ABI5* activation	Qi et al., 2020
		PYL8/PYL9 —‖ PIF4 → *ABI5*		
PIF3 PIF5	*PYL3, PYL6, PYL12, SnRK2.2, ABI5* and *ABF3*	Potential targets indicated (further study of gene expression required)	Unknown	Liang et al., 2020
FHY3	*ABI1, ABI3, ABI4* and *ABI5*	DET1 —‖ FHY3 → *ABI1*, *ABI3*, *ABI4* and *ABI5*	Activation of the ABA pathway after the dark-to-light transition. Pathway repressor: DET1	Xu et al., 2020
DET1	*ABI5*	DET1 —‖ *ABI5* (*via* recruiting HDA6 to *ABI5* promoter)	Positive regulation of light-induced greening; epigenetic control of *ABI5* expression	Xu et al., 2020
CO	*ABI3*	CO —‖ ABI3	Quiescence in developing seedlings, rapid induction of flowering	Kurup et al., 2000
phyB	SnRK2.6	SnRK2.6-phyB	Negative role of SnRK2.6 in red light-induced stomatal opening	Li et al., 2021
FCA	ABI5	FCA → ABI5	Adaptation of embryo and flower development to stress-induced ROS, heat, cold, and drought conditions	Lee et al., 2015
LHY	*ABI1, ABI2*	LHY —‖ *ABI1/2*	Coupling between the circadian clock and ABA pathways	Adams et al., 2018
	*SnRK2.2*	LHY —‖ *SnRK2.2*	Activation of drought-tolerance processes	
	*ABF1*	LHY —‖ *ABF1*	Potentiation of ABA responses in the morning	
	*ABI5*	LHY → *ABI5*	Responses to unexpectedly hot or dry conditions in the daytime	
FLC	ABI5	SnRK2s → ABI5 (and/or other ABFs) → *FLC*	Inhibitory effect on floral transition	Wang et al., 2013
	ABI4	ABI4 → *FLC*		Shu et al., 2016

Our analysis showed that most of the described interactions between the light signaling system and ABA are aimed at regulating ABA signaling from the side of light signals. First of all, this is mainly the regulation of the expression of *ABI5*, as well as *ABI1*, *ABI3* and *ABI4* genes, which is carried out using the following modules: HY5 → *ABI5*, BBX21—‖ *ABI5*, phyB —‖ PIF1 → *ABI3* and *ABI5*, ELF3 —‖ *PIF4* → *ABI5*, DET1 —‖ FHY3 → *ABI1*, *ABI3*, *ABI4* and *ABI5*, DET1 —‖ *ABI5*, and LHY → *ABI5*. LHY suppresses the expression of *ABI1/ABI2*, *SnRK2.2*, and *ABF1*. Protein–protein interactions are realized through CO —‖ ABI3 and FCA → ABI5.

From the ABA side, the light signaling system is regulated by the ABA receptors PYL8/PYL9, which promote PIFs protein accumulation in the dark. SnRK2.6 interacts with phyB, having a negative role in red light-induced stomatal opening. ABI5 and ABI4 enhance the expression of the *FLC* gene.

## Effect of Light on the Expression of Genes Encoding ABA Signaling Components

A global analysis of gene expression during intense light treatment showed that ABA levels were gradually increased from 0.5 to 72 h due to enhanced expression of ABA biosynthetic genes (Huang et al., 2019). The authors suggested the important role of ABA in middle- and long-term high light stress response, although the data on the gene expression of the ABA signaling network were somewhat surprising. Among the down-regulated genes were genes encoding ABA receptors: *PYR1*, *PYL3*, *PYL4*, *PYL5*, *PYL7,* and *PYL9*. PP2Cs genes were upregulated, such as *ABI1*, *ABI2*, *HAB1*, *AHG1*, *PP2CA*, and especially *HAI1*, *HAI2,* and *HAI3* (Huang et al., 2019). *SnRK2.9*, *ABF2*, and *ABF3* were slightly downregulated with different dynamics. Altogether, these data indicate silencing of ABA signal transmission. It is not yet known which mechanisms regulate the expression of the listed genes. A recent work by Qi et al. (2020) contributed to this problem, as they showed that PIFs physically interact with the ABA receptors PYL8 and PYL9. These authors showed that PYL8 and PYL9 negatively regulate PIF4-mediated trans-activation of *ABI5*. Liang et al. (2020) analyzed the DNA-binding sites for PIF3 and showed that several ABA signaling genes were found to be potential targets, such as *PYL3*, *PYL6*, *PYL12*, *SnRK2.2*, *ABI5,* and *ABF3* (Table 1).

The complexity of the ABA–light interaction was noticed by Oh et al. (2009) who found that responsiveness to ABA is due to the summed activity of both positively and negatively regulated genes of the ABA signaling pathway. The view that the ABA–light collaboration is a complex and finely tuned process was also supported by Qi et al. (2020).

## HY5-Based Modules

HY5 is a transcription factor that promotes photomorphogenesis in light, acting downstream of the light receptor network and directly activating the transcription of light-induced genes. Among these are genes encoding transcription factors, signaling proteins, and flavonoid biosynthetic enzymes (Gangappa and Botto, 2016; Cañibano et al., 2021). PhyB-dependent induction of HY5 is well known (Guo et al., 2021). The activation of HY5 by phyB may take place directly by physical interaction (Chai et al., 2015) or through intermediaries (Ortigosa et al., 2020). HY5 forms a complex network with proteins of the light signaling system, such as COP1, PIF3, FHY3, FAR1, HFR1, light-mediated development protein DET1, and B-box binding (BBX) proteins (BioGrid annotation).

The important effect of cooperation between ABA and light is the decision to transition from a dormant seed to photoautotrophic growth. It is well documented that the interaction of light signaling components with ABA signaling is necessary to fine-tune seed germination and seedling development, and this interaction occurs *via* signaling on the *ABI5* promoter (Chen et al., 2008). HY5 binds to the *ABI5* promoter and activates its expression, thus mediating ABA responses in seed germination, seedling growth, and root development (Chen et al., 2008). HY5 is an important hub in the crosstalk between light and cold response pathways, and an integrator of ABA and ROS signaling (Wang et al., 2018). HY5 is a positive regulator of cold acclimation; in particular, increased expression of *HY5* induces increased expression of cold-responsive (*COR*) genes (Catalá et al., 2011). The authors proposed a model in which HY5 would promote the full development of cold acclimation, integrating low temperature and light signaling. According to this model, HY5 is stabilized by cold by protein stabilization through the nuclear depletion of COP1 (Catalá et al., 2011). HY5 also induces several genes involved in anthocyanin biosynthesis (Gangappa and Botto, 2016).

The current model suggests that ROS production and scavenging pathways are integrated into a signal transduction network, which is necessary for stress acclimation (Mittler et al., 2011; Devireddy et al., 2020). In tomato, ABA signaling realized through *ABI5* induces H_2_O_2_ production *via* the activation of respiratory burst oxidase homolog 1 (SlRboh1; Zhou et al., 2014; Wang et al., 2018). This mechanism includes binding HY5 to the *ABI5* promoter and ABI5-inducing *RBOH1* expression (Wang et al., 2018), establishing the signaling sequence HY5 → *ABI5* → *Rboh1*. A similar mechanism is likely to function in *Arabidopsis*. Therefore, through binding to the promoter of *ABI5*, HY5 triggers enhanced photoprotection through the induction of an apoplastic H_2_O_2_ burst that influences antioxidant status, cyclic electron flux, and nonphotochemical quenching. Improved photoprotection allows shaded leaves to better tolerate photoinhibition. The HY5-ABI5 pathway is necessary to ensure tolerance to excess light and cold (Gangappa and Botto, 2016).

HY5 and its homolog HYH, collectively with PIF1 and PIF3 form a dynamic activation-suppression transcriptional module HY5/HYH-PIF1/PIF3 to establish an appropriate cellular redox status in the response to light (Chen et al., 2013; Toledo-Ortiz et al., 2014). PIF1/PIF3 and HY5/HYH function antagonistically; they physically interact and jointly regulate the expression of ROS-responsive genes (Chen et al., 2013). The author’s model shows that in the dark, PIF1 and PIF3 accumulate while HY5 and HYH are degraded. In high light conditions, photosensitized Protochlorophyllide (Pchlide) generates singlet oxygen, which causes photooxidative cell death. However, light promotes the stabilization of HY5/HYH and PIF1/PIF3 degradation, causing activation the ROS signaling and the expression of ROS responsive genes, thus adjusting ROS level and allowing plants to survive under light stress conditions (Chen et al., 2013). Besides HY5/HYH-PIF1/PIF3 module-mediated control of ROS-responsive genes, these genes can be controlled by HY5 and other PIFs (PIF4 and PIF5) in association with circadian clock components (Toledo-Ortiz et al., 2014). HY5, PIF4, and PIF5 proteins are under strong diurnal regulation, while PIF1 and PIF3 are not rhythmic (Toledo-Ortiz et al., 2014). The link between HY5/PIF modules and the ABA signaling system is realized through regulation of *ABI3* and *ABI5* expression: HY5 → *ABI5,* PIF1 → *ABI3/ABI5,* and PIF4 → *ABI5* (Table 1).

## Signaling on the ABI5 Promoter

According to TAIR annotation, ABI5 participates in the ABA-activated signaling pathway, negative regulation of seed germination, positive regulation of transcription, responses to chitin, gibberellin, salt stress, water deprivation, seed development, seed germination, and the sugar-mediated signaling pathway. *ABI5* encodes the basic leucine zipper transcription factor involved in ABA signaling. The *Arabidopsis* ABA-insensitive *abi5* mutants have pleiotropic defects in ABA response, including altered expression of ABA-regulated genes and decreased sensitivity to ABA inhibition of germination. ABI5 regulates a subset of late-embryogenesis-abundant genes during seed development and vegetative growth.

**Figure 3:**
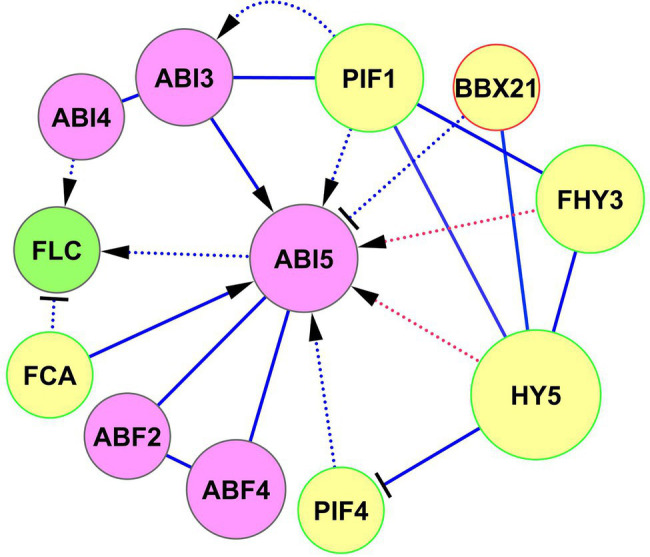
Crosslinking of ABA and light signaling on ABI5. Solid lines represent protein–protein interactions presented in PAIR, BioGRID, and IntAct, and dotted lines represent transcriptional regulation. ABA-responsive element binding factors of the ABA signaling network, such as ABF2, ABF4, and ABI3 (involved in abiotic stress defense and stress memory) cooperate with ABI5 to jointly regulate its function. Light signaling proteins PIF1, PIF4, FHY3/FAR1, and HY5 activate the expression of *ABI5*, whereas BBX21 inhibits its expression. FCA activates ABI5 by protein–protein interaction and jointly with ABI5 regulates expression of *FLC*. FLC and FCA are proteins that substantially reduce plant water use and are important for heat and cold adaptation. Red activator lines from FHY3/FAR1 and HY5 indicate cooperative effects HY5, DET1, and FHY3 signaling on ABI5 function (see also details in Figure 4). Abbreviations: ABI3,4,5, ABA insensitive 3,4,5; ABFs, abscisic acid responsive elements-binding factors; PIFs, phytochrome-interacting transcription factors; HY5, transcription factor HY5 (protein long hypocotyls 5); BBX21, B-box zinc finger transcription factor BBX21; FHY3/FAR1, far-red elongated hypocotyls 3/protein far-red impaired response 1; FCA, flowering time control protein; FLC, MADS-box protein flowering locus C.

G-box *cis*-elements of the *ABI5* promoter are binding sites for HY5, BBX21, and PIF1, whereas FHY3 and FAR1 bind FBS cis-elements (CACGCGC) on *ABI5* (Xu et al., 2014; Ma and Li, 2018). BBX21, also known as STH2, is a BBX protein, which binds to the *ABI5* promoter and negatively regulates *ABI5* expression (Xu et al., 2014; Kang et al., 2018). In the BBX protein subfamily, BBX21/STH2 and BBX22/STH3 (also known as LZF1) function as positive regulators of photomorphogenesis, whereas BBX18/DBB1a, BBX19/DBB1b, BBX24/STO, and BBX25/STH1 are negative regulators (Xu et al., 2014). Two light signaling proteins, HRB2 (CHD3-type chromatin-remodeling factor PICKLE) and BBX21 repress ABA signaling by *ABI5* suppression to sustain gas exchange in drought conditions (Kang et al., 2018). Mutations in *HRB2* or *BBX21* cause ABA hypersensitivity and reduced water loss (Kang et al., 2018). Accordingly, plants overexpressing *BBX21* showed reduced sensitivity to ABA and improved photosynthetic capacity under drought conditions (Gómez-Ocampo et al., 2021). Interactions of ABI5 with the ABA–light signaling network are shown in Figure 3. The interaction of HY5, DET1, and FHY3 signaling with ABI5 is described in more detail in the next section.

## FHY3/FAR1 and DET1 in the Process of Interaction with ABA Signaling

Far-red elongated hypocotyl (FHY3) and far-red impaired response 1 (FAR1) are positive regulators of ABA signaling because they bind to the *ABI5* promoter and activate its transcription, thus mediating abiotic stress responses and ABA signal transduction (Tang et al., 2013; Ma and Li, 2018). Disruption of FHY3 and/or FAR1 decreases the sensitivity to ABA-mediated inhibition of seed germination, seedling development, and primary root growth (Tang et al., 2013). The *fhy3* and *far1* mutants have wider stomata, lose water faster, and are more sensitive to drought than the wild type. FAR1 negatively regulates light-induced ROS accumulation and oxidative stress-induced cell death by activating the transcription of *myo-inositol-1-phosphate synthase 1* (*MIPS1*; Ma and Li, 2018).

In addition to its well-known function in plant growth and development during the vegetative stage, FHY3 plays an essential role in floral meristem determinacy and shoot apical meristem maintenance (Li et al., 2016). FHY3 directly represses CLAVATA 3 in shoot apical meristem, and consequently regulates WUSCHEL to maintain the stem cell pool. FHY3 mainly acts as a transcriptional repressor in flower development, in contrast to its activator role in seedlings, i.e., its function varies in different organs (Li et al., 2016). Why FHY3 acts as either activator or repressor in different organs or developmental stages remains to be studied (Ma and Li, 2018).

**Figure 4:**
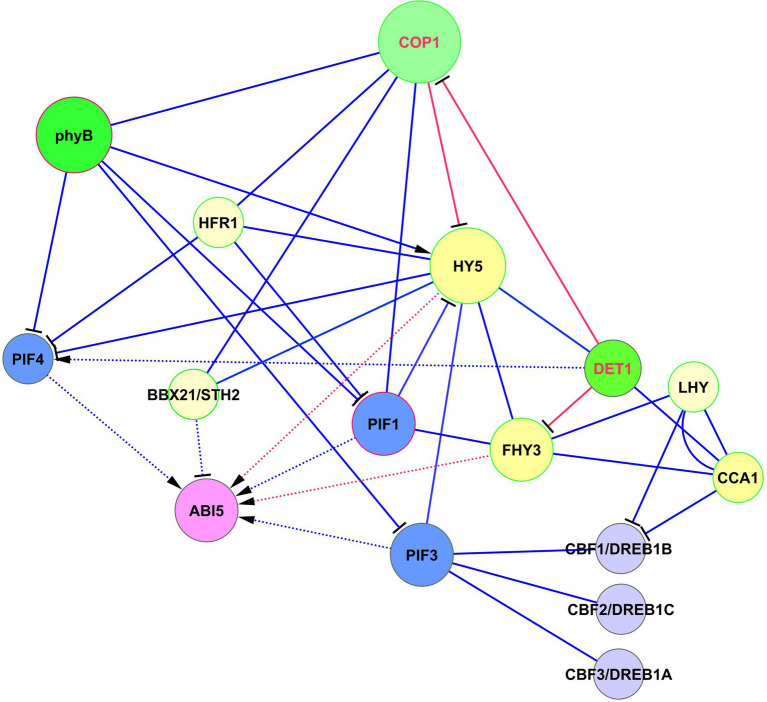
Crossing of HY5, DET1, and FHY3 signaling with ABI5 function. *ABI5* expression is regulated in two ways. The first one represents activation of *ABI5* expression by HY5 by the DET1 —‖ COP1 —‖ HY5 → *ABI5* module. The second level of regulation is realized by parallel pathway *via* DET1 —‖ FHY3 → *ABI5* and DET1/COP1 → PIF4 → *ABI5* modules, where DET1/COP1 association promotes *PIF4* expression and stabilizes PIF4 protein. DET1 also can form a complex with the CCA1/LHY module. Abbreviations: ABI5, ABA insensitive 5; phyA/B, phytochromes phyA and phyB; PIFs, phytochrome-interacting transcription factors; HY5, transcription factor HY5; FHY3, far-red elongated hypocotyls 3; BBX21, B-box zinc finger transcription factor BBX21; DET1, light-mediated development protein; CCA1, CCA1, circadian clock associated 1; LHY, late elongated hypocotyl; HFR1, transcription factor HFR1; COP1, E3 ubiquitin-protein ligase COP1; CBFs/DREBs, dehydration-responsive element-binding proteins.

The role of FHY3 in the light-ABA signaling is complex and depends on the activity of the light-mediated development protein DET1 (DET1), which is an important modulator of the light signaling system (Xu et al., 2020). During seedling development, DET1 physically interacts with FHY3 and represses FHY3-mediated activation of ABA-responsive genes. Among these genes are *ABI1*, *ABI3*, *ABI4,* and *ABI5* (Xu et al., 2020). The authors tested these genes as markers of the ABA-activated pathway. Interestingly, while ABI3, ABI4, and ABI5 are transcription factors, ABI1 is a protein phosphatase. ABI5 acts as a central hub for these interactions (Yadukrishnan and Datta, 2021). The DET1 —‖ FHY3 → *ABI5* pathway acts only in light conditions. The role of this pathway is that after the dark-to-light transition, ABA induces high expression of *ABI5*. This response is balanced and adjusted by DET1 through FHY3 repression that causes the greening of etiolated seedlings and increases their adaptation to light. COP1 and PIFs were proposed could act collectively with DET1 and FHY3 to prevent the activity of the signaling module DET1 —‖ FHY3 → *ABI5* in darkness (Xu et al., 2020). The crossing of HY5, DET1, and FHY3 signaling with *ABI5* is presented in Figure 4.

The important role of DET1 is its involvement in epigenetic control, suggesting a role in the generation of stress memory. DET1 physically interacts with histone deacetylase 6 (HDA6), recruiting HDA6 to the *ABI5* promoter (Xu et al., 2020). This inhibits the activation of *ABI5* by regulating the enrichment of H3K27ac and H3K4me3 modifications. This mechanism can be independent of FHY3 and contributes to the stress memory.

DET1 is also involved in ABA responses by interacting with ABI3 (Kurup et al., 2000). The consequences and outputs of this interaction are yet unknown. Because DET1 and COP1 are repressors of photomorphogenesis and repressors of multiple light responses, destruction of any of these proteins results in the induction of de-etiolation in darkness and hyper-photomorphogenic phenotype in the light. DET1 is associated with COP1 *in vivo* (Cañibano et al., 2021), and DET1/COP1 and HY5 are now considered as two main distinct signaling components (albeit with some overlap of function) in the regulation of thermosensory elongation (Xu et al., 2020). The DET1/COP1 association promotes *PIF4* expression and stabilizes PIF4 protein (Gangappa and Kumar, 2017, 2018). The author’s model suggests that the DET1/COP1 → PIF4 signaling sequence acts as an important module for the control of growth and plant stress defense in response to seasonal signals. They showed that DET1/COP1-activated PIF4 binds to the promoters of growth genes and activates their expression, whereas HY5 negatively regulates thermosensory growth by competing with PIF4. Elevated temperatures decrease HY5 activity and activate PIF4 target genes (Gangappa and Kumar, 2017).

DET1, *via* interaction with COP1, decreases the abundance of HY5, thus avoiding hyper-photomorphogenic responses (Cañibano et al., 2021). These authors proposed an interesting mechanism according to which these proteins control HY5 abundance. They postulated that DET1-mediated COP1 degradation is necessary to maintain COP1 turnover and activity. Indeed, COP1 is a short-lived protein with a high turnover rate. By down-regulating HY5 levels, DET1 prevents HY5 binding to primary targets. Since HY5 behaves in light conditions mainly as a transcriptional activator, DET1’s function is necessary to prevent HY5 over-accumulation that causes misregulation of numerous downstream genes. HY5 transcriptional activity over a limited gene number can be regulated by titration of its availability (Cañibano et al., 2021), resembling the process known from animal studies, where signaling molecules form spatiotemporal concentration gradients that respond to a wide range of signal intensities, the so-called digital process (Tay et al., 2010). Thus, DET1 —‖ FHY3 → *ABI5* and DET1/COP1 → PIF4 → *ABI5* modules emerge as important players in the regulation of development and stress reactions, in addition to HY5-based modules.

## LHY/CCA1 as a Link Between the Circadian Clock and Stress Response

LHY and CCA1 bind to the same region of gene promoters, but they are only partially redundant (UniProtKB annotation). The CCA1 protein responds to environmental light and temperature signals and, therefore, may maintain circadian rhythms as well as adapting plants to the environment (Zhang et al., 2021).

Adams et al. (2018) suggested a mechanism for the circadian control of ABA accumulation in plants. ABA accumulates during water deficit, reaching its peak in the evening. In the morning, LHY inhibits the expression of ABA biosynthetic genes encoding 9-cis-epoxycarotenoid dioxygenase enzymes that decreases ABA level and ensures the rhythmic accumulation of ABA. LHY also controls ABA signaling pathway by inhibiting the expression of the *ABI1*, *ABI2*, *SnRK2.2*, and *ABF1* but activating the expression of *ABI5* (Adams et al., 2018). The resulting effect of LHY is that under physiologically relevant conditions, LHY acts to potentiate ABA-dependent stress responses by promoting the expression of ABA-responsive genes involved in establishing increased tolerance to drought and osmotic stress.

Although LHY and CCA1 are almost identical within their DNA-binding domains, they have functional differences. The most represented motif for LHY and CCA1 is the EE motif (AAATATCT or AGATATTT). However, the ABRE motif (ABA-specific motif) was over-represented only in LHY-specific target promoters, but not in CCA1-specific target promoters (Adams et al., 2018). Commenting on this article, Belbin and Dodd (2018) noted that there is some overlap in the roles of LHY and CCA1 in regulating ABA signaling. It is interesting that DET1 forms a complex with LHY/CCA1 to repress two-component response regulator-like APRR1/TOC1 expression in the morning (Paik et al., 2017), which adds an extra level of regulation to the above described DET1 —‖ FHY3 → *ABI5* and DET1/COP1 → PIF4 → *ABI5* modules.

Thus, both LHY and CCA1 transcription factors, acting in conjunction with the ABA signaling system, regulate the adaptation of plants to cold, high temperature, lack of water, and osmotic stress, thereby optimizing defense responses under circadian rhythms.

## HFR1’s Role in Shade Avoidance and Dark-Induced Senescence

An important player in shade avoidance and dark-induced senescence is transcription factor HFR1 (long hypocotyl in FR light1), which is the non-DNA binding atypical HLH factor (Rolauffs et al., 2012; Pham et al., 2018b; Ueda et al., 2020). Like FHY3, HFR1 is involved in FR light signal transduction and physically interacts with phyA (IntAct and STRING databases). According to GO annotation, HFR1 is involved in the ABA-activated signaling pathway. However, HFR1 does not interact with any component of the ABA signaling pathway but interacts with PIF1, PIF3, PIF4, and PIF5 (BioGRID). Probably, the involvement in the ABA signaling pathway occurs *via* PIFs. PIF4 and PIF5 are required for shade avoidance and dark-induced senescence acting as positive regulators, while HFR1 inhibits these responses (Hornitschek et al., 2009). PIF4 and PIF5 support shade avoidance syndrome by directly binding to G-boxes in promoters of shade marker genes, but their action is limited in the shade when HFR1 accumulates. Since PIF4 and PIF5 act upstream of ABA signaling (Ueda et al., 2020) by directly activating the expression of *ABI5* (Sakuraba et al., 2014), the signaling sequence is as follows: HFR1 —‖ PIF4/PIF5 → *ABI5* (Figure 4).

Likewise, *via* interference with PIF4 by binding to promoters of genes that function in cell elongation, HFR1 regulates cell elongation in response to high temperatures (Ikeda et al., 2021). PIF1 and HFR1 undergo mutual degradation in the dark. Under red and far-red light, HFR1 is stabilized by phytochrome-mediated inhibition of COP1-SPA. The increased abundance of HFR1 sequesters PIF1 and other PIFs to promote seed germination and seedling de-etiolation under light (Xu et al., 2017; Pham et al., 2018b). DET1 suppresses seed germination by destabilizing HFR1 and stabilizing PIF1 (Shi et al., 2015).

In post-germination seedling development, low R:FR conditions induce the accumulation of ABA and modulate the expression of several ABA biosynthetic and signaling genes (Yadukrishnan and Datta, 2021). ABA induced in response to shade inhibits the shade-triggered hypocotyl elongation by the action of ABI3 and ABI4 (Ortiz-Alcaide et al., 2019). HFR1 does not interact with transcription factors ABI3 and ABI4 (BioGRID). Therefore, the role of HFR1 in ABA-induced hypocotyl elongation remains to be studied.

## FCA/FLC in Flower Development and Thermal Adaptation

The RNA-binding protein FCA is a component of flowering pathways in *Arabidopsis* and a regulator of the *FLC* gene, which encodes MADS-box protein FLOWERING LOCUS C (Tian et al., 2019). FCA interacts with ABI5 and is essential for the proper expression of ABI5-regulated genes involved in antioxidant defense and thermotolerance (Lee et al., 2015). FCA not only regulates the function of many genes involved in adaptation to stress-induced ROS, heat, cold, and drought conditions *via* FLC and ABI5 but also adjusts the function of protective genes by itself through chromatin modification and RNA metabolism (Lee et al., 2015).

In turn, signaling components of ABA affect *FLC* expression, thereby influencing floral transition in *Arabidopsis*. ABA signaling causes the inhibitory effect on floral transition by stimulating the SnRK2s → ABI5 (and/or other ABFs) → *FLC* pathway (Wang et al., 2013). Direct binding of ABI5 to the ABRE/G-box promoter elements in *FLC* was demonstrated by chromatin immunoprecipitation. Shu et al. (2016) showed that ABI4, an important protein in the ABA signaling network, negatively regulates floral transition by directly activating *FLC* transcription. Interestingly, the authors characterized the observed effect as the “tip of the iceberg” and suggested that further discoveries will be made in this area.

Histone acetylation is important in the FCA-mediated thermal adaptation of developing seedlings, chlorophyll biosynthesis, and seedling photosynthesis (Ha et al., 2017). The FLC/FCA module also functions in cold conditions, providing adaptation to winter temperatures through an *FLC* antisense transcript *COOLAIR* (Jiao et al., 2019; Tian et al., 2019). It has been shown that FCA interacts with SWI3A and SWI3B, components of the Switch/Sucrose non-fermenting, ATP-dependent chromatin remodeling complex (SWI/SNF CRC; Sarnowski et al., 2005), suggesting the role the chromatin-based control on FCA function. The signaling sequence FCA → ABI5 → *FLC* (Figure 3) is interesting and needs further examination, especially concerning brassinosteroid signaling and acclimation processes (Bulgakov and Avramenko, 2020; Jing and Lin, 2020). Thus, the FLC/FCA module is regulated both by the ABA pathway and influences the ABA components, and these two signaling systems jointly coordinate the processes of flower development under different temperature conditions.

## ELF3: Circadian System Compensation Against Daily and Seasonal Changes

ELF3 encodes a nuclear protein that is expressed rhythmically and interacts with phyB to regulate plant development and flowering. The important role of this regulator, as a component of the ELF3-ELF4-LUX ARRHYTHMO (LUX) evening complex, is to modulate light input to the circadian clock and connect light signaling with thermotolerance. ELF3 negatively regulates the activity of *PIF4* at high temperatures (Sakuraba et al., 2014; Kim et al., 2020), establishing the ELF3-*PIF4*-*ABI5* signaling module. As a result, PIF4 accelerates leaf senescence at high ambient temperature by increasing the expression of the major senescence-promoting NAC transcription factor *ORESARA1* with the participation of ABA and ethylene signaling. Although GO annotation indicates that ELF3 is involved in the ABA signaling, the direct interaction of ELF3 (as well as ELF4 and LUX) with ABA signaling components has not been described.

Recent studies of rhythmic chromatin modifications revealed a role of chromatin remodeling factors in ELF3 functioning (Zhao et al., 2021). For instance, histone deacetylase 9 (HDA9) interacts with ELF3 (Lee et al., 2019; Park et al., 2019) and can be considered as an early regulator of thermomorphogenesis. HDA9 is also can interact with many transcription factors, including HY5, ABI3, and ABI4, thus contributing to the formation of HDA9-dependent epigenetic states regulating growth, acclimation, photoperiodic flowering, senescence, aging, dormancy, and germination (de Rooij et al., 2020). Likewise, SWI/SNF chromatin remodelers interact with many players of the light-ABA network, such as the ABA-related protein phosphatases ABI1, ABI2, HAB1, and PP2CA, SnRK2s, the ABA-related transcription factors ABI3, ABF1, and ABF3 as well as proteins from the light signaling system: LUX, CO and CONSTANS-like proteins, FCA, the transcription factor SPATULA, and others (Bulgakov et al., 2019). Thus, epigenetic interactions in the ABA–light signaling system represent a promising topic related to the establishment of local epigenetic landscapes.

## Light, Cold Tolerance, and SnRK2s

**Figure 5:**
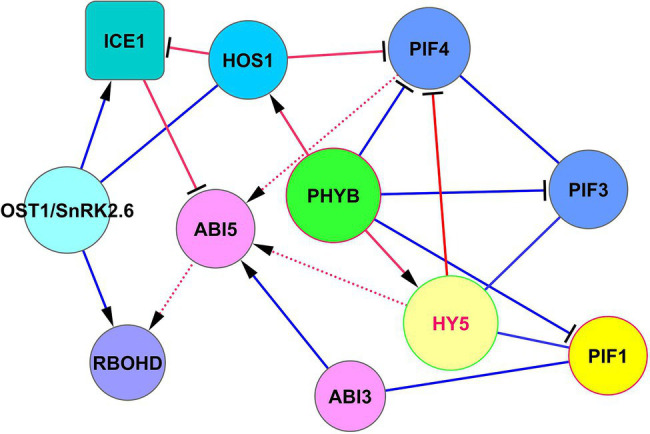
The intersection of ICE1 and HY5 functions in cold adaptation. ABI5 serves as a concentrator of ICE1, PIF4, and HY5 signaling. ABI5 receives signals from these proteins to regulate *RbohD* expression and H_2_O_2_ level to establish cold protection. Note, that HY5 does not physically interact with PIF4 (BioGRID) but regulates its function through competitive chromatin binding to PIF4 targets (Gangappa and Kumar, 2017). ICE1 physically associates with ABI5 and antagonizes its transcriptional function by concurring with G-box type *cis* elements in promoters of responsive genes (Hu et al., 2019). Abbreviations: ICE1, inducer of CBP expression 1; HOS1, E3 ubiquitin-protein ligase HOS1; ABI3/5, ABA insensitive 3/5; OST1/SnRK2.6, SNF1-related protein kinase 2.6; PIFs, phytochrome-interacting transcription factors; PHYB, phytochrome phyB; HY5, transcription factor HY5. RBOHD, respiratory burst oxidase homolog protein D (NADPH oxidase RbohD).

Above we discussed that HY5 can support the development of cold acclimation, joining cold and light signaling (Section “HY5-Based Modules”). Transcription factor ICE1 (inducer of CBF expression 1) represents another important protein in establishing cold resistance. The intersection of the signaling pathways of these cold resistance factors is of great interest for bioengineering purposes. The SnRK2.6/OST1-HOS1-ICE1 signaling module controls cold tolerance *via* the CBF-*COR* cold signaling pathway (Ding et al., 2015). In this module, OST1 activates ICE1, and the pleiotropic regulator HOS1 (E3 ubiquitin-protein ligase; synonym: high expression of osmotically responsive genes 1) is necessary to adapt plant development both to short-term cold stress and freezing tolerance by interacting with ICE1 (Dong et al., 2006; Ding et al., 2015; Ye et al., 2019). In *hos1* mutants, ICE1 is not degraded (Dong et al., 2006), which leads to the acquisition of cold tolerance *via* the unified ICE-CBF pathway (Chinnusamy et al., 2003; Kim et al., 2015). ICE1 induces *CBF1*, *CBF2,* and *CBF3* by binding to the gene promoters (Kim et al., 2015). The module HOS1 —‖ ICE1 ← OST1 is shown in Figure 5. HOS1 not only inhibits ICE1 but also physically interacts with PhyB and CO and suppresses the function of PIF4 (Kim et al., 2017), thereby linking cold response and photoperiodic response (Lazaro et al., 2015). Therefore, HOS1 is controlled by the ABA signaling *via* SnRK2.6/OST1, and by light signaling *via* phyB. This creates a question about crossing these two important pathways.

HOS1 and HY5 do not interact physically (BioGRID). The intersection of their signaling occurs *via* ABI5, as indicated in Figure 5. Rapid local and systemic stomatal responses of *Arabidopsis* to intense light are dependent on the function OST1/SnRK2.6 (Devireddy et al., 2020). These responses are dependent on ABA-derived ROS signaling by the OST1/SnRK2.6 → RbohD pathway (Devireddy et al., 2020). The process of cold acclimation can be realized by RbohD activation at the transcriptional and posttranscriptional levels through ABI5 → *RbohD* and SnRK2.6 → RbohD, thus increasing H_2_O_2_ levels, which is necessary to establish cold protection and tolerance to excess light (Zhou et al., 2012; Wang et al., 2018; Devireddy et al., 2020). Considering ROS production as an important factor for cold and high-intensity light acclimation, Miller et al. (2009) and Mittler et al. (2011) showed that RbohD plays a key role in these processes.

The resulting signaling modules for the HOS1 role in these processes can be written as HOS1 —‖ ICE1 —‖ ABI5 → *RbohD* and HOS1 —‖ PIF4 → *ABI5* → *RbohD*, which have an overlap in ROS regulation *via ABI5* function (Figure 5).

**Figure 6:**
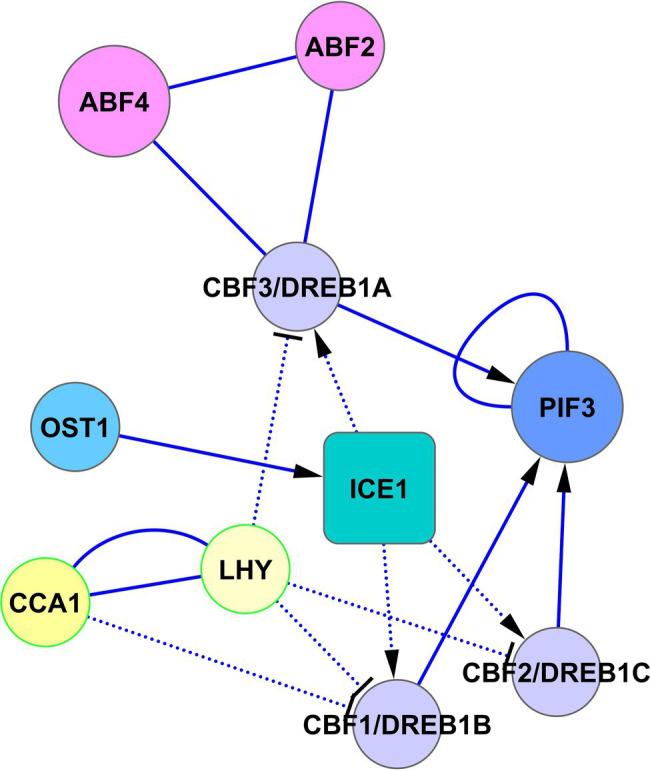
Regulation of CBF/DREB1 pathway by ABA and light signaling. The activated CBF-cold-responsive (*COR*) cold-signaling pathway increases cold tolerance. Transcription of *CBF1/DREB1B*, *CBF2/DREB1C*, and *CBF3/DREB1A* is regulated oppositely by ABA signaling *via* the cold-activated SnRK2.6-ICE1 pathway and light signaling *via* CCA1/LHY. CCA1 and LHY suppress the expression of DREB1s under unstressed conditions and are rapidly degraded in response to cold (Kidokoro et al., 2021), allowing activation of the CBF-*COR* cold signaling pathway. In turn, CBF proteins, accumulated in response to cold, interact with PIF3 and prevent PIF3 degradation with subsequent de-repression of *COR* genes. Abbreviations: ABFs, abscisic acid responsive elements-binding factors; PIF3, transcription factor PIF3; OST1/SnRK2.6, SNF1-related protein kinase 2.6; ICE1, inducer of CBP expression 1; CCA1, circadian clock associated 1; LHY, protein late elongated hypocotyl; CBFs/DREBs, dehydration-responsive element-binding proteins.

Another way of cold adaptation is the signal crossing between PIF3, CBF1, CBF2, and CBF3 (Figure 6). Jiang et al. (2020) showed that PIF3 interacts with CBF1, CBF2, and CBF3 proteins. They proposed a model in which CBF proteins, accumulated in response to cold, interact with PIF3, and this interaction prevents PIF3 and phyB degradation. Cold-stabilized phyB initiates the degradation of PIF1, PIF4, and PIF5, which allows the de-repression of *COR* genes. Collectively, these data mean that *CBF1*, *CBF2,* and *CBF*3 genes are regulated by SnRK2.6-ICE1 and possibly by ABF2 and ABF4 from the side of the ABA signaling, as well as PIF3 and LHY/CCA1 from the side of the light signaling (Figure 6).

Until recently, the interaction of SnRK2s with components of the light signaling network remained elusive. Li et al. (2021) reported the interaction between SnRK2.6 and phyB and showed a negative role of OST1/SnRK2.6 in stomatal opening induced by red light. A *snrk2.6* mutant exhibited a significantly larger stomatal aperture under red light treatment, while overexpression lines exhibited smaller stomatal apertures (Li et al., 2021). This observation places ABA signaling, mediated through SnRK2.6, upstream of phyB signaling.

## Concluding Remarks

The rapidly growing number of articles on the association of ABA and light signaling is driven by the emerging prospects of plant growth management in changing environmental conditions. Regulation of plant development in greenhouses is also important because the ability to manipulate spectral characteristics with LED sources would help combine growth control and stress tolerance. Surprisingly, analysis of protein interaction networking combined with analysis of the literature revealed a relatively small number of interacting modules (Table 1).

These modules are at different stages of understanding their functioning. If crossing of ABA and light signaling on the *ABI5* promoter, ABA-associated HY5- and FHY3/FAR1-based modules relatively well worked out, then LHY/CCA1, ELF3, and FCA modules require further in-depth study. Likewise, the functional importance of ABI3-CO, ABF4-FT, and ABI3-TOC1 interactions needs further examination. Interaction of important hubs in ABA and light signaling systems, such as SnRK2.6 and phyB, is beginning to be studied and appears to be promising.

During writing this work, we drew attention to the growing body of articles on the epigenetic regulation of the ABA–light signaling system. Indeed, the study of stress memory of plants, a phenomenon through which information on a past stress cue is retained and results in a modified response upon recurring stress, promises great perspectives in the regulation of plant growth, since chromatin regulators instruct time-dependent control of transcription. Concerning the ABA–light signaling system, this topic will undoubtedly be further developed in the coming years.

Summarizing the information described, we would like to highlight several key points.

- As before, ABI5 is the main protein that binds ABA and light signaling. Recent research has added a new level of complexity to the process mediated by the HY5-ABI5 interaction, since HY5 itself is under complex control. New modules have been added such as DET1 —‖ FHY3 → ABI5 and DET1/COP1 → PIF4 → ABI5, and a new look is now needed to assess the overall picture of the impact of light signaling components on ABA signaling mediated through ABI5 to fully understand the logic of cooperation.
- The influence of class 3 sucrose nonfermenting-1-related protein kinases (SnRK2s) on the light signaling system is an important new topic.
- Interaction of ABA receptors with PIFs, because this new topic directly links early ABA signaling to light signaling.
- Part of the research that deals with ROS, as more and more data point to the role of ROS in ABA–light cooperation.
- The FCA → ABI5 → *FLC* signaling sequence is also a key, because FCA/FLC system is now under deep investigation and FCA/FLC affect ABA signaling and ABA signaling affects FCA/FLC. By itself, FLC significantly reduces plant water use because it binds to multiple target genes involved in the response to water shortage. In turn, FCA regulates the function of many genes involved in adaptation to stress-induced ROS, heat, cold, and drought through FLC and ABI5, and regulates the function of defense genes through chromatin modification.
- Chromatin modifications, because many of the ABA signaling components and light signaling components are under the control of chromatin modifiers. There are currently no review articles describing the ABA–light interaction from this point of view, but new experimental data are constantly being added.

## Author Contributions

VB contributed to conception, data analysis, and manuscript writing. OK performed analysis and interpretation of data, revising for important intellectual content, and final approval. All authors contributed to the article and approved the submitted version.

## Funding

Financial support was provided by the Russian Science Foundation, Grant No. 20-16-00016 (VB).

## Conflict of Interest

The authors declare that the research was conducted in the absence of any commercial or financial relationships that could be construed as a potential conflict of interest.

## Publisher’s Note

All claims expressed in this article are solely those of the authors and do not necessarily represent those of their affiliated organizations, or those of the publisher, the editors and the reviewers. Any product that may be evaluated in this article, or claim that may be made by its manufacturer, is not guaranteed or endorsed by the publisher.

## Footnotes

^1^ http://www.cls.zju.edu.cn/pair/

^2^ http://thebiogrid.org/

^3^ https://www.uniprot.org/

^4^ https://www.arabidopsis.org/

^5^ https://www.ebi.ac.uk/intact/interactors/
